# Acetalax (Oxyphenisatin Acetate, NSC 59687) and Bisacodyl Cause Oncosis in Triple-Negative Breast Cancer Cell Lines by Poisoning the Ion Exchange Membrane Protein TRPM4

**DOI:** 10.1158/2767-9764.CRC-24-0093

**Published:** 2024-08-14

**Authors:** Makito Mizunuma, Christophe E. Redon, Liton Kumar Saha, Andy D. Tran, Anjali Dhall, Robin Sebastian, Daiki Taniyama, Michael J. Kruhlak, William C. Reinhold, Naoko Takebe, Yves Pommier

**Affiliations:** 1 Developmental Therapeutics Branch, Center for Cancer Research, National Cancer Institute, National Institutes of Health, Bethesda, Maryland.; 2 Laboratory of Cancer Biology and Genetics, Center for Cancer Research, National Cancer Institute, National Institutes of Health, Bethesda, Maryland.; 3 Division of Cancer Treatment and Diagnosis, National Cancer Institute, National Institutes of Health, Bethesda, Maryland.

## Abstract

Triple-negative breast cancer (TNBC) is clinically aggressive and relatively unresponsive to current therapies. Therefore, the development of new anticancer agents is needed to satisfy clinical needs. Oxyphenisatin acetate (Acetalax), which had been used as a laxative, has recently been reported to have anticancer activity in murine models. In this study, we demonstrate that Acetalax and its diphenolic laxative structural analogue bisacodyl (Dulcolax) exhibit potent antiproliferative activity in TNBC cell lines and cause oncosis, a nonapoptotic cell death characterized by cellular and nuclear swelling and cell membrane blebbing, leading to mitochondrial dysfunction, ATP depletion, and enhanced immune and inflammatory responses. Mechanistically, we provide evidence that transient receptor potential melastatin member 4 (TRPM4) is poisoned by Acetalax and bisacodyl in MDA-MB468, BT549, and HS578T TNBC cells. MDA-MB231 and MDA-MB436 TNBC cells without endogenous TRPM4 expression as well as TRPM4-knockout TNBC cells were found to be Acetalax- and bisacodyl-resistant. Conversely, ectopic expression of TRPM4 sensitized MDA-MB231 and MDA-MB436 cells to Acetalax. TRPM4 was also lost in cells with acquired Acetalax resistance. Moreover, TRPM4 is rapidly degraded by the ubiquitin–proteasome system upon acute exposure to Acetalax and bisacodyl. Together, these results demonstrate that TRPM4 is a previously unknown target of Acetalax and bisacodyl and that TRPM4 expression in cancer cells is a predictor of Acetalax and bisacodyl efficacy and could be used for the clinical development of these drugs as anticancer agents.

**Significance:**

Acetalax and bisacodyl kill cancer cells by causing oncosis following poisoning of the plasma membrane sodium transporter TRPM4 and represent a new therapeutic approach for TNBC.

## Introduction

Triple-negative breast cancer (TNBC), which does not express estrogen receptor, progesterone receptor, and HER2 (*ERBB2*, Erb-B2 receptor), represents an imperative therapeutic need due to its highly invasive nature and relatively poor response to existing treatments (1). New treatments are highly desirable for patients with relapsed/refractory TNBC, as there is no standard chemotherapy regimen. Responses to treatment are usually short in duration and followed by rapid relapse, with visceral and brain metastases common. Available therapies for patients with advanced TNBC include antimetabolites, capecitabine and gemcitabine; a nontaxane microtubule inhibitor, eribulin; and DNA cross-linker platinums. However, the median progression-free survival with chemotherapy ranges from 1.7 to 3.7 months.

**Figure 1:**
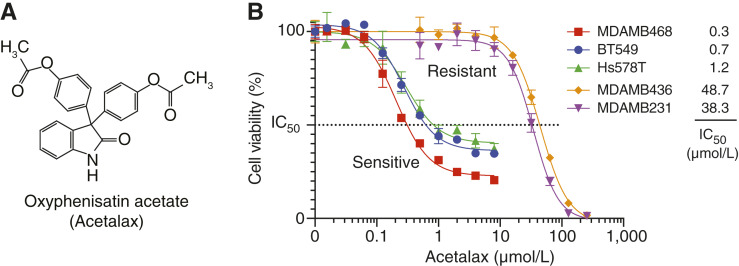
Acetalax structure and its effect on TNBC cell lines. **A,** Two-dimensional structure of Acetalax (NSC 59687). **B,** Cell viability assays for five TNBC cells measured at 72 hours using CellTiter-Glo 2. Experiments were performed in triplicate (*n* = 3). Bars represented SD.

The development of new anticancer agents remains an unmet goal. Repurposing previously FDA-approved drugs offers an alternative, which can save time and cost as their dosage, safety, and side effect information is already known. Oxyphenisatin acetate (NSC 59687), commonly called Acetalax, is a diphenyl oxindole originally developed as a laxative in the 1950s (Fig. 1A; refs. 2, 3). Recently, Acetalax and its laxative analogue bisacodyl (Dulcolax; Supplementary Fig. S1A) have been reported to have anticancer activity in prostate and ovarian cancer models, as well as some breast cancer models (2, 4). Additionally, we recently reported that Acetalax displays a unique profile of activity across the 1,000 cell lines of the Sanger Institute [Genomics of Drug Sensitivity in Cancer (GDSC) database] and observed its selective activity in the 22 TNBC cell lines of the GDSC database (5). Although the drug has been hypothesized to trigger a starvation response in cancer cell lines, the detailed mechanism of its anticancer effect remains to be established (2).

A correlative study of gene expression and drug sensitivity using approximately 17,000 bulk RNA sequencing (RNA-seq) samples from The Cancer Genome Atlas and Genotype-Tissue Expression databases suggested a correlation between transient receptor potential melastatin member 4 (TRPM4) expression and sensitivity to Acetalax (6). However, causality and mechanistic studies were not performed. TRPM4 is a nonselective membrane pore channel protein for cations, which can be activated by an increase in either intracellular Ca^2+^ or ATP depletion (7–9). When activated, TRPM4 induces an influx of extracellular Na^+^ and water into the cell. However, TRPM4 is insensitive to increases in intracellular Na^+^ concentration, so continued rapid Na^+^ influx can contribute to oncosis in stroke (10). Additionally, TRPM4 may be implicated in regulating cancer cell migration, controlling cytoskeleton maintenance, and preventing epithelial–mesenchymal transition (EMT; refs. 6–9).

The goals of the present study were to establish the activity of Acetalax in TNBC and to elucidate Acetalax’s molecular mechanism(s) of action. To achieve this goal, we selected three highly sensitive and two resistant TNBC cell lines and examined their differential morphologic changes: (i) cellular swelling, (ii) nuclear swelling, (iii) membrane blebbing, (iv) cellular membrane permeability changes, (v) mitochondrial membrane potential shift, (vi) reduction in cellular ATP, and (vii) mitochondrial fragmentation. We demonstrate that all of these changes occur in a drug sensitivity–dependent manner and are reminiscent of oncosis, a nonapoptotic cell death (11, 12) characterized by cellular swelling, organelle swelling, blebbing, and increased membrane permeability (10, 12, 13).

To elucidate the molecular target of Acetalax, we first correlated the sensitivity of 710 different cancer cell lines with gene expression and identified TRPM4 as a correlate. We also generated Acetalax-resistant clones by chronic exposure (CE) to Acetalax and examined gene expression changes by RNA-seq. We established TRPM4 knockout (KO) and complemented cell lines and investigated the causal relationship between Acetalax and TRPM4 expression.

## Materials and Methods

### Cell lines and reagents

Human TNBC cell lines MDA-MB468 (RRID: CVCL_0419) and MDA-MB436 (RRID: CVCL_0623) were obtained from ATCC. ATCC performs authentication and quality-control tests on all distribution lots of cell lines. We did not repeat this authentication. Hs578 and MDA-MB231 (RRID: CVCL_0062) cell lines were obtained from Dr. Stan Lipkowitz, NIH/NCI, with cell line identification done on February 10, 2023 (Laragen sequencing and genotyping). The BT549 (RRID: CVCL_1092) cell line was obtained from the Developmental Therapeutics Program (DTP). The DTP has its own internal authentication, and we did not duplicate this. MDA-MB468, MDA-MB231, MDA-MB436, and BT549 cells were cultured in RPMI medium (11875093, Gibco) containing 10% FBS (100106, GeminiBio) and 1% penicillin/streptomycin (15140-122, Gibco). Hs578T cells were grown in DMEM (11965092, Gibco) containing 10% FBS (GeminiBio) and 1% penicillin/streptomycin (Gibco). Cells were cultured at 37°C in 5% CO_2_ and passed approximately 15 to 20 times each. Cells were monitored for *Mycoplasma* using the MycoAlert Mycoplasma Detection Kit (LT07701 Lonza) approximately every 10 weeks, with the last tests done around September 15, 2024. Acetalax (NSC 59687) was obtained from the NCI, DTP. MG132 (C2211) was purchased from MilliporeSigma (474790).

### Cell viability assay

Cells were plated in 384-well plates at a density of 300 cells/well in 30 μL medium. After 24 hours, the cells were treated with various concentrations of Acetalax (see figure legends for details). After treatment for 72 hours, cell viability was measured by CellTiter-Glo 2.0 Luminescent Cell Viability Assay (Promega) on a SpectraMax i3x microplate reader (Molecular Devices). Data were analyzed using GraphPad Prism 9.0 software (GraphPad Software Inc.)*.* Experiments were performed in triplicate (*n* = 3).

### Immunofluorescence microscopy

Cells were plated on 22 × 22-mm microscope cover glasses in a 6-well plate at a density of 1 to 3 × 10^5^ cells/well in 2 mL of a complete medium. After 24 hours, the cells were treated with Acetalax as described in the figure legends. After washing with PBS, the cells were fixed in 4% paraformaldehyde and permeabilized with 0.3% Triton X-100/PBS and blocked with 3% BSA/PBS-T for 1 hour. The cells were stained with anti-TOMM20 antibody (HPA011562, Sigma-Aldrich, RRID: AB_1080326; 1:250 dilution) PBS-T overnight at 4°C. After washing with PBS, the cells were incubated with Alexa Fluor 488 phalloidin antibody (A12379, Invitrogen; 1:200 dilution), PBS-T, and Alexa Fluor 568 secondary antibody (1:1,000 dilution) for 1 hour at room temperature. After that, the cells were incubated with 4',6-diamidino-2-phenylindole (DAPI; 62248, Thermo Fisher Scientific; 1:1,000 dilution) for 30 minutes and mounted with ProLong Glass Antifade Mountant (P36980, Invitrogen) on cover slides. Images were taken using a Nikon SoRa spinning disk confocal microscope (magnification, 40× or 60×).

### Cell and nuclear size analyses and blebbing cell frequency analysis

Cell and nucleus size analyses and bleb cell frequency analysis were performed with photographs taken under identical conditions using a Nikon SoRa spinning disk confocal microscope. Photographs were taken at arbitrary locations with a 40× objective lens and contained 2 to 40 cells per field of view. These data were read using ImageJ2 (ver. 2.14) software to recognize size. The number of bleb cells was also counted manually. Cells that partially protruded from the image were excluded from the analysis, and cells that were adherent to each other were manually separated using ImageJ2 to accurately measure the size of a single cell.

### Cell membrane permeability test

Cells were seeded in 6-well plates at a density of 3 × 10^5^ cells per well. After overnight incubation, the cells were incubated with treatments at indicated concentrations for 5 minutes. The cells were harvested by trypsinization, and the pellets were resuspended in propidium iodide (PI) solution (P3566, Thermo Fisher Scientific; 20 μg/mL) for 5 minutes. The samples were then subjected to flow cytometry using a FACS Canto (Becton Dickinson) with 10^4^ acquisitions in the PI channel. Data were analyzed using FlowJo software version 10.8.2 (Becton Dickinson).

### Mitochondrial membrane potential assay

Mitochondrial membrane potential was evaluated using the fluorescent probe MT-1 MitoMP Detection Kit (MT13, Dojindo) according to the protocol. Briefly, cells (3 × 10^5^ cells/well) were seeded for 24 hours in the medium in 6-well plates and treated with 1 μmol/L Acetalax for various times. Carbonyl cyanide-*p*-trifluoromethoxyphenylhydrazone treatment at 2 μmol/L for 1 hour was used as a positive control. Cells were incubated in the medium containing 1,000× diluted working solution for 30 minutes and then treated with drugs, washed with PBS, and harvested by trypsinization. Imaging buffer solution was added, and the samples were subjected to flow cytometry.

### ATP level determination

In brief, cells were plated in 384-well white plates at a density of 3,000 cells/well in 30 μL medium. After 24 hours, the cells were treated with Acetalax at 1 μmol/L for various times (detailed treatment conditions are described in figure legends). After treatment, the intracellular ATP level was measured by CellTiter-Glo 2.0 Luminescent Cell Viability Assay (Promega) on a SpectraMax i3x microplate reader (Molecular Devices). The ATP depletion rate was measured by the luminosity decrease of the treated group as compared with the untreated group, each done in triplicate (*n* = 3). The formula used to determine the ATP depletion rate was

ATP depletion (%)= (1 – Luminossity with drug/Luminossity of control) × 100

### Mitochondrial fragment analysis

Three-dimensional image analysis was performed using Imaris version 9.9.0 (Oxford Instruments). Mitochondrial surfaces were generated from TOMM20 staining using the Surfaces plugins combined with the Labkit machine learning plugin software (ver.0.3.11; ref. 14). Geometric statistics from mitochondrial surfaces were collected, and surfaces were classified as fragmented or filamentous based on volume and sphericity of the individual surfaces. We defined that the fragmented mitochondria in the cells were 0.1 to 1 μm^3^ in volume and >0.6 in sphericity for MDA-MB468; 0.1 to 5 μm^3^ in volume and >0.6 in sphericity for BT549; 0.1 to 0.6 μm^3^ in volume and >0.7 in sphericity for Hs578T; 0.1 to 1 μm^3^ in volume and >0.6 in sphericity for MDA-MB436; and 0.1 to 0.6 μm^3^ in volume and >0.7 in sphericity for MDA-MB231. The percent cumulative fragmented volume was calculated as the sum of the fragmented surface volume divided by the total mitochondrial volume per cell. We collected the amount of mitochondria in approximately 30 cells in each TNBC cell and analyzed whether there was a difference in the amount of fragmented mitochondria between the Acetalax-untreated and -treated (10 μmol/L, 4 hour) groups.

### Western blot

Cell pellets were collected and lysed with NETN300 buffer [1% NP40, 300 mmol/L NaCl, 0.1 mmol/L EDTA, and 50 mmol/L Tris (pH 7.5)] supplemented with protease inhibitor cocktail (Cell Signaling Technology). After determining protein concentrations by Bio-Rad Protein Assay Dye Reagent (Bio-Rad), cell lysates were separated by reducing SDS-PAGE gels (Invitrogen) and transferred onto polyvinylidene difluoride membranes (Millipore). The membranes were immunoblotted with the following antibodies: TRPM4 (OTI10H5; ab123936, Abcam, RRID: AB_10976061; 1:1,000 dilution, and GAPDH (6C5; sc-32233, Santa Cruz Biotechnology; 1:2,000 dilution). After overnight incubation, the membranes were incubated with species-appropriate horseradish peroxidase–conjugated secondary antibodies. Protein signals were visualized using the ChemiDoc MP Imaging System (Bio-Rad) with SuperSignal West Pico PLUS or Ultimate Sensitivity Chemiluminescent Substrate (Thermo Fisher Scientific, 34580 or A38555).

Using Western blotting to measure the degradation of TRPM4, lysing buffer was not used, and the agent-treated cells were collected by trypsin, added to PBS, and treated with an equal volume of SDS buffer with a final concentration of 5% β-mercaptoethanol, and heated at 95°C for 5 minutes after sonication. Subsequent procedures were performed as for other Western blots.

### Generating Acetalax-resistant cells

We created Acetalax-resistant cells by chronically exposing MDA-MB468 and BT549, which were originally sensitive. A total of 3 × 10^5^ cells were seeded in a 10-cm dish, and when the dish reached almost full confluency in 1 to 2 weeks, the cells were passaged with double the concentration of Acetalax. Initially, the Acetalax concentration was started at 0.2 μmol/L. When the Acetalax concentration was increased to 51.2 μmol/L, the cells stopped growing. Therefore, cells chronically exposed to 40 μmol/L Acetalax were designated as Acetalax CE cells and are Acetalax-resistant clones. CE cells were used in each experiment after a 2-week Acetalax-free period.

### Generation of TRPM4 knockout in human BT549 and MDA-MB468 cells

We used CRISPR/Cas9-mediated genome editing to generate TRPM4 knockout (KO) in BT549 and MDA-MB468 cell lines. TRPM4-KO cells were generated by targeting exon 2 of the *TRPM4* gene. The sequences of guide RNA (gRNA) for *TRPM4* gene disruption are 5′-GTC​AAC​TAT​GAA​CGT​CGT​GCA​GG-3′ and were chosen using CRISPR direct software. For cloning, the gRNA was inserted into the BbsI site of pX459 (Cat# 48139, Addgene). pX459 expresses gRNA under the control of the U6 promoter and Cas9 under the chicken β-actin promoter. pX459-gRNA was then transfected into BT549 and MDA-MB468 cells, which were seeded in a 6-cm dish containing ∼ 60% confluency using Lipofectamine 3000 transfection reagent (Cat# L3000001, Thermo Fisher Scientific) according to the manufacturer’s instructions. Twenty-four hours after the transfection, puromycin was added to a final concentration of 2 μg/mL. The cells were further incubated for 48 hours with the puromycin-containing medium. After removing puromycin, we incubated the cells for approximately 2 weeks to isolate the single clones. The gene-disruption events were confirmed by Western blotting analysis.

### RNA-seq

RNA-seq was done for MDA-MB468 and BT549 parental cells and CE cells that had been Acetalax-free for 2 weeks. RNA was extracted from a control group of these cells and from cells treated with Acetalax at 1 μmol/L for 6 hours. RNA was extracted as per protocol using PureLink RNA Mini Kit (Cat: 12183018A, Invitrogen). The extracted RNA was measured for RNA integrity number to confirm that there were no problems with RNA quality. We used 800 ng of total RNA samples to generate the library (PolyA selected). The kits used for the library preparation were NEBNext PolyA mRNA Magnetic Isolation Module (NEB #E7490), NEBNext Ultra II Directional RNA Library Prep Kit for Illumina (NEB #E7760), and NEBNext Multiplex Oligos for Illumina (96 unique dual-index primer pairs, NEB, E6440) as described in the manufacturer’s manual. Sequencing was carried out in the Illumina NextSeq 2000 instrument using the NextSeq 2000 P2 kit (200 cycles) with 101 × 101 paired-end configuration. The Gene Expression Omnibus repository accession number is GSE268793.

### Bioinformatic analysis

Next-generation sequencing data were generated for both BT549 and MDA-MB468 cells (control vs. Acetalax-treated) with or without CE conditions. RNA-seq fastq files were aligned with the hg19 reference genome using STAR aligner version 2.7.10b (PMID: 23104886). Aligned reads were sorted and indexed by Samtools (v 1.17). The “–quantMode GeneCounts” option of STAR was used to calculate read counts per gene for each condition, and fragments per kilobase of transcript per million mapped reads (FPKM) values per gene were calculated using the “convertCounts” R package. After that, genes having zero expressions under both the conditions were removed from the downstream analysis. FPKM values were normalized using log_2_ after the addition of “1” as a constant for each condition. A score was computed for each gene by taking the difference between (treatment and control) for each condition. These genes were ranked based on the score; positive and negative scores represent upregulation and downregulation of genes under a particular condition, respectively. Enrichment analysis was performed using the GSEApy Python library. We used 50 hallmark gene sets (15), EMT gene sets (16), immediate early response genes (17), and immune signatures (18) to perform enrichment analysis.

### Generation of TRPM4 knock-in in human MDA-MB231 and MDA-MB436 cells

Transfection of the human FLAG-tagged TRPM4 WT expression plasmid (OriGene, Cat#RC216888) was carried out using Lipofectamine 3000 reagents (Cat#L3000015, Thermo Fisher Scientific) according to the manufacturer’s protocol. The expression of TRPM4 was verified by Western blot using anti-TRPM4 and anti-FLAG antibodies both at 72 hours after transfection, followed by cell viability assay as described above.

### Statistical analysis

Statistical analyses were performed using GraphPad Prism 9.0 software (GraphPad Software Inc.). The Mann–Whitney *U* test was performed for cell and nucleus size analyses and mitochondrial fragmentation analysis, and the *χ*^2^ test was used for blebbing frequency analysis. Dose–response curves were plotted, and IC_50_ values were calculated using GraphPad Prism 9.0. Statistical significance was set at *P* < 0.05.

The Acetalax activity data versus TRPM4 scatter plot for five TNBC cell lines were plotted using the R Project for Statistical Computing version 3.8.6 (RRID: SCR_001905). The Acetalax activity data versus TRPM4 scatter plot for 22 GDSC TNBC cell lines were done using CellMinerCDB (https://discover.nci.nih.gov/rsconnect/cellminercdb/) using “Univariate Analysis\Plot Data” tabs choosing “Select Tissues\To include\Breast Triple Negative” to limit the cell lines (19).

### Data availability

The drug activities and transcript data used in the scatter plots are available at CellMinerCDB. To generate these scatter plots, please choose Univariate Analysis\Plot Data\GDSC-MGH-Sanger cell line set. Data are restricted to TNBC using select tissues to include breast triple-negative.

## Results

### Acetalax (NSC 59687) and bisacodyl sensitivity in TNBC cell lines

Because Acetalax was consistently more potent than bisacodyl, while having a very similar activity pattern, across the multiple cancer cell lines tested (Fig. 1A and B; Supplementary Figs. S1A–S1D), we focused our molecular pharmacology and mechanistic studies primarily on Acetalax and only extended the main findings for bisacodyl. Based on our prior observations in a wide range of cancer cell lines from different tissues of origin such as NCI60 (NCI 60 cell lines) consisting of 710 cell lines of the GDSC (Supplementary Fig. S1; ref. 5), we here focused on TNBC cell lines because of their resistance to the clinically available anticancer drugs. The viability of cells exposed to Acetalax was first tested in five different TNBC cell lines (Fig. 1B) selected from the GDSC database for their differential response to Acetalax (see Supplementary Fig. S1D; ref. 5). After 72 hours of treatment with different drug concentrations, we confirmed that three of the TNBC cell lines, MDA-MB468, BT549, and Hs578T (IC_50_ 0.33, 0.72, and 1.19 μmol/L, respectively), are highly sensitive to Acetalax, whereas the other two TNBC cell lines, MDA-MB436 and MDA-MB231 (IC_50_ 48.7 and 38.4 μmol/L, respectively), showed 40- to 120-fold resistance to Acetalax (Fig. 1B). For bisacodyl, the same cell lines showed the most and least sensitivity (Supplementary Fig. S1).

### Acetalax induces morphologic changes consistent with oncosis

Since its discovery more than a century ago, oncosis has been identified as a cell death mode characterized by cellular swelling (11, 13). This is in contrast with apoptosis, which is characterized by cell shrinkage and the formation of apoptotic bodies (11, 20). To determine whether Acetalax induces changes in cellular morphology that could be assigned to a specific type of cell death, we examined whether Acetalax induces morphologic changes in the five TNBC cell lines with differential drug responses (Fig. 1B).

**Figure 2:**
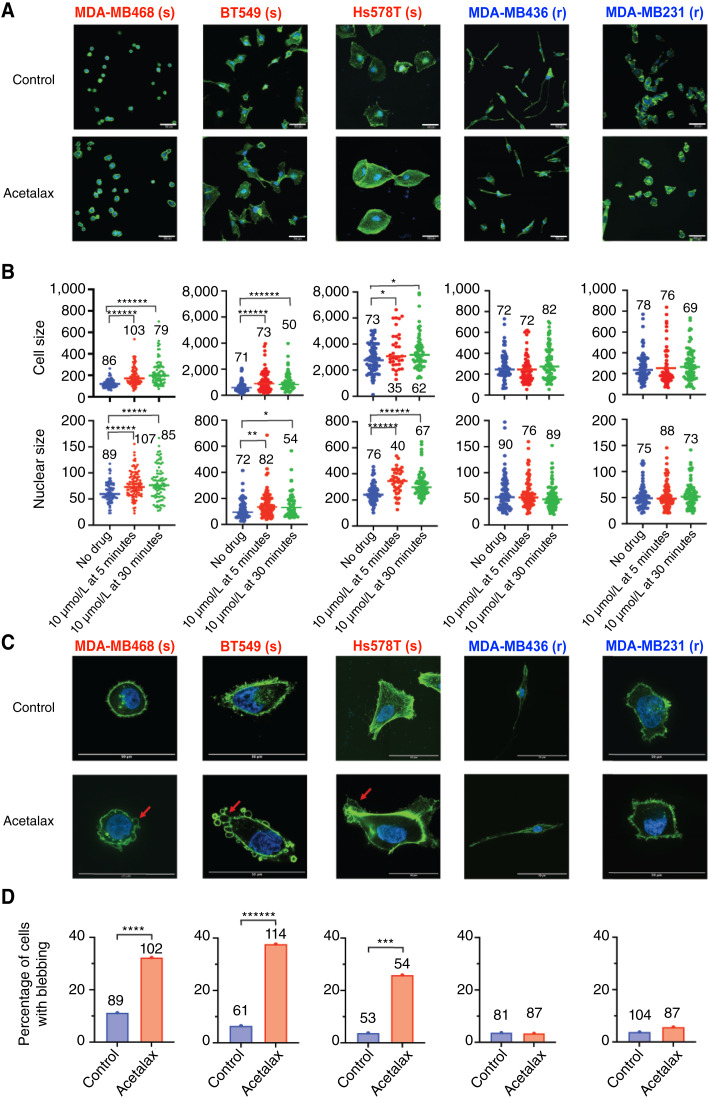
Morphologic changes caused by Acetalax in TNBC cell lines with differential sensitivity. **A,** Representative microscopy images of cell lines treated with Acetalax (10 μmol/L for 30 minutes) and stained in blue with DAPI (binds DNA) and green with phalloidin (stains actin). Control, without Acetalax treatment. Scale bars, 50 μm. (s), most Acetalax-sensitive cell lines; (r), most Acetalax-resistant cell lines (Fig. 1B). **B,** Top, Violin plots for the quantitation of Acetalax-induced cell size increase at 5 and 30 minutes. The numbers represent the number of cells analyzed. The lines indicate medians. Each dot is a cell. MDA-MB468, BT549, and Hs578T have significant changes in cell size. Bottom, Violin plots for the quantitation of Acetalax-induced nuclear size increase at 5 and 30 minutes. Each dot is a nucleus. The numbers represent the number of nuclei analyzed. Nuclear size increase between the control and Acetalax-treated cells significantly for MDA-MB468, BT549, and Hs578T. **C,** Microscopy of membrane blebbing caused by Acetalax (10 μmol/L for 30 minutes). Control, untreated cells; red arrows, cell membrane blebs. The images are chosen to fill the frames for improved visibility without normalization by scale. Scale bars, 50 μm. **D,** Quantitation of membrane blebbing in multiple cells as shown in **C**. In the most sensitive (s) cell lines, MDA-MB468, BT549, and Hs578T, Acetalax treatment significantly increased the frequency of bleb cells. Statistical significance: *, *P* < 0.05; **, *P* < 0.01; ***, *P* < 0.005; ****, *P* < 0.001; *****, *P* < 0.0005; ******, *P* < 0.0001 (using the Mann–Whitney *U* test, calculated in PRISM 9.0).

Representative images for the five TNBC cell lines treated with Acetalax for 30 minutes are shown in Fig. 2A. All these images were taken under the same conditions. Quantitative analyses of cellular and nuclear sizes for multiple cells as defined by two-dimensional microscopy at 5- and 30-minute treatments with Acetalax are shown in Fig. 2B. The three Acetalax-sensitive cells, MDA-MB468, BT549, and Hs578T, showed an increase in overall size of the cells and nuclei within 5 minutes of drug treatment. Conversely, both cell and nuclear sizes of the resistant MDA-MB436 and MDA-MB231 cells remained unchanged. High-resolution pictures of representative Acetalax-treated cells also showed plasma membrane blebbing in the most sensitive MDA-MB468, BT549, and Hs578T cells, as shown by the red arrows in Fig. 2C. The frequency of blebbing cells was quantified and found significantly increased in the Acetalax-sensitive but not the Acetalax-resistant cell lines (Fig. 2D).

These results indicate that Acetalax causes both cell and nuclear swelling, as well as plasma membrane blebbing within 5 to 30 minutes after treatment. Similar results were observed with bisacodyl (Supplementary Fig. S2A–S2C), suggesting the rapid induction of oncosis by Acetalax and bisacodyl.

### Acetalax causes cell membrane permeability increase, mitochondrial membrane potential decrease, ATP depletion, and mitochondrial fragmentation

**Figure 3:**
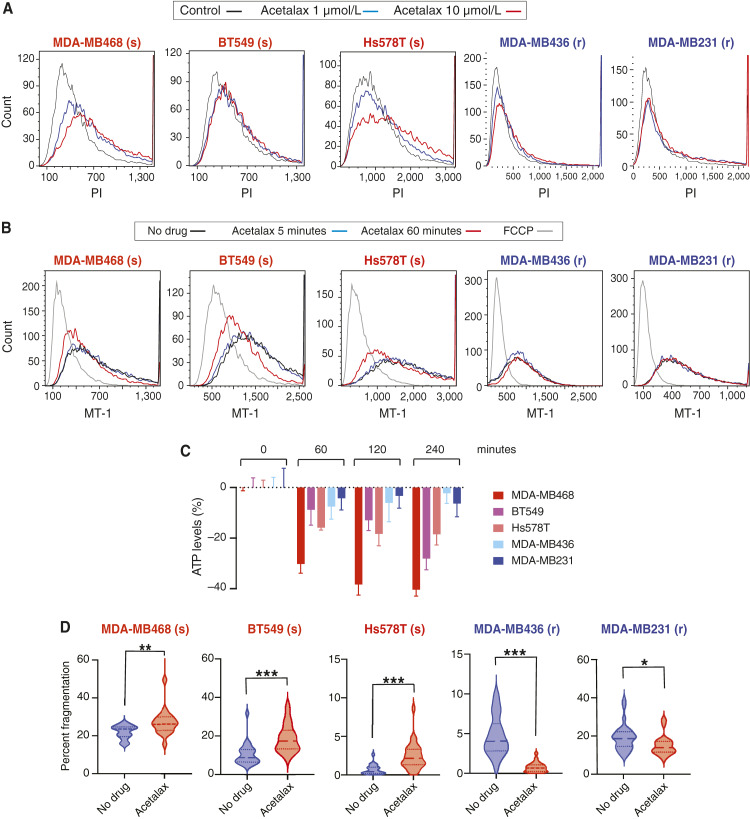
Plasma membrane permeabilization and mitochondrial changes caused by Acetalax. **A,** Representative flow cytometry data showing plasma membrane permeabilization induced by Acetalax within 5 minutes as measured by PI assays. Acetalax concentrations are color-coded as indicated. The *x*-axis shows PI absorbance, and the *y*-axis shows cell counts. The right shifts of the peaks in the MDA-MB468, BT549, and Hs578T sensitive cell lines indicate increased permeability. **B,** Flow cytometric mitochondrial membrane potential (MT-1) assay. Acetalax treatments were at 1 μmol/L for 5 and 60 minutes. Carbonyl cyanide-*p*-trifluoromethoxyphenylhydrazone (2 μmol/L for 60 minutes) was used as the positive control. The *x*-axis shows mitochondrial membrane potential, and the *y*-axis shows cell counts. The left shifts of the peaks seen in the sensitive cell lines indicate decreased mitochondrial membrane potential. Carbonyl cyanide-*p*-trifluoromethoxyphenylhydrazone, an uncoupler of oxidative phosphorylation, was used as the positive control. **C,** ATP depletion. Cells were treated with Acetalax 60, 120, and 240 minutes at 1 μmol/L. ATP depletion was calculated as the percentage change compared with the untreated group as measured by CellTiter-Glo 2. **D,** Mitochondrial fragmentation. Cells were treated with Acetalax for 4 hours at 10 μmol/L. Fragmentation volume percentage was calculated as the total fragmented surface volume divided by the total mitochondrial volume per cell. Percentage of fragmented mitochondria on the *y*-axis represents the comparison between control and Acetalax-treated groups. **A,** Significant change in mitochondrial fragmentation is indicated by the brackets. *, *P* < 0.01; **, *P* < 0.005; ***, *P* < 0.0001 (using the Mann–Whitney *U* test, calculated in Prism 9.0). Center lines indicate medians. FCCP, Carbonyl cyanide-*p*-trifluoromethoxyphenylhydrazone.

In oncosis, increased permeability of the cell membrane is a known cause of cell swelling and blebbing (10). Therefore, we investigated the effect of Acetalax on cell membrane permeability using the dye exclusion test with PI, a fluorescent intercalating agent that permeates cells with compromised cell membranes. Figure 3A shows that Acetalax produced a concentration-dependent increase in intracellular PI as early as 5 minutes after drug treatment in the drug-sensitive TNBC cell lines MDA-MB468, BT549, and Hs578T, indicating acute cell membrane permeabilization. In contrast, there was no detectable membrane permeability change in the resistant MDA-MB436 and MDA-MB231 cell lines (Fig. 3A).

Because mitochondrial dysfunction and ATP depletion have also been reported during oncosis (11–13), we examined whether Acetalax causes mitochondrial dysfunction and ATP depletion. Mitochondrial dysfunction was determined as a decrease in mitochondrial membrane potential measured by the MT-1 assay. In the Acetalax-sensitive cell lines, MT-1 decreased at 60 minutes exposure to Acetalax (Fig. 3B). In contrast, there was no change in the resistant cells (Fig. 3B). These results demonstrate that Acetalax induces mitochondrial dysfunction in the sensitive cell lines following changes in cell permeability.

Next, we examined ATP depletion, as measured by CellTiter-Glo 2 Assay in the Acetalax-treated versus untreated cells. As shown in Fig. 3C, the Acetalax-sensitive cell lines showed more than 10% ATP depletion at 60 minutes and 20% to 40% ATP depletion after 4 hours of treatment. In contrast, the resistant cell lines showed only 2% to 10% ATP depletion after 4 hours of Acetalax exposure (Fig. 3C).

We also examined mitochondrial morphology in cells treated with Acetalax. We classified mitochondria based on their size and sphericity and defined small and nearly spherical mitochondria as fragmented mitochondria. Mitochondrial changes, as shown in Supplementary Fig. S3 (2D image), were measured using 3D images. The percentage of fragmented mitochondria significantly increased in the Acetalax-treated sensitive but not in the resistant cell lines at 4 hours in association with significant fusions (Fig. 3D).

Together, these results show that Acetalax induces a loss of intracellular ATP, mitochondrial morphologic alterations and a decrease in mitochondrial membrane potential subsequent to the rapid increase in cell and nuclear sizes and the permeabilization of the plasma membrane.

### TRPM4 expression is causally related to Acetalax sensitivity

To discover the molecular target of Acetalax, we adopted an unbiased approach and used the large drug response dataset of the GDSC-MGH-Sanger in which we had tested both Acetalax and bisacodyl responses (Supplementary Fig. S1; ref. 5). Using the “Compare Pattern” tool (tab) of CellMinerCDB (https://discover.nci.nih.gov/; ref. 21) and looking for correlations between Acetalax activity and gene expression measured by RNA-seq across the whole genome, we found that across the cell lines tested for Acetalax response, TRPM4 expression had the highest correlation with Acetalax activity of 37,263 transcripts (Pearson correlation coefficient = 0.50; *P* value = 1.7e−19; Supplementary Fig. S4). To reproduce Supplementary Fig. S4, go to CellMinerCDB and choose the Univariate Analysis tab, GDSC-MGH-Sanger for cell line set for both axes. For the *x*-axis, choose drug activity as the data type and Acetalax as the identifier. For the *y*-axis, choose RNA-seq as the data type and TRPM4 as the identifier. To reproduce the highest correlation result, use CellMinerCDB’s Comparative Pattern tool and select Univariate Analysis\Compare Patterns. For both axes, again choose GDSC-MGH-Sanger data. For the *x*-axis, choose drug activity as the data type and Acetalax as the identifier. For the “select molecular or activity data,” choose molecular data. In the “data type,” enter “xsq,” which is our abbreviation for RNA-seq. It shows that the top gene of 37,263 entries is TRPM4. This finding is consistent with our observations that Acetalax induces a rapid increase in both cell size and membrane permeability (Figs. 2 and 3) and with the fact that *TRPM4* encodes a prominent membrane permeability pore gene mediating the transport of monovalent cations across membranes (22).

**Figure 4:**
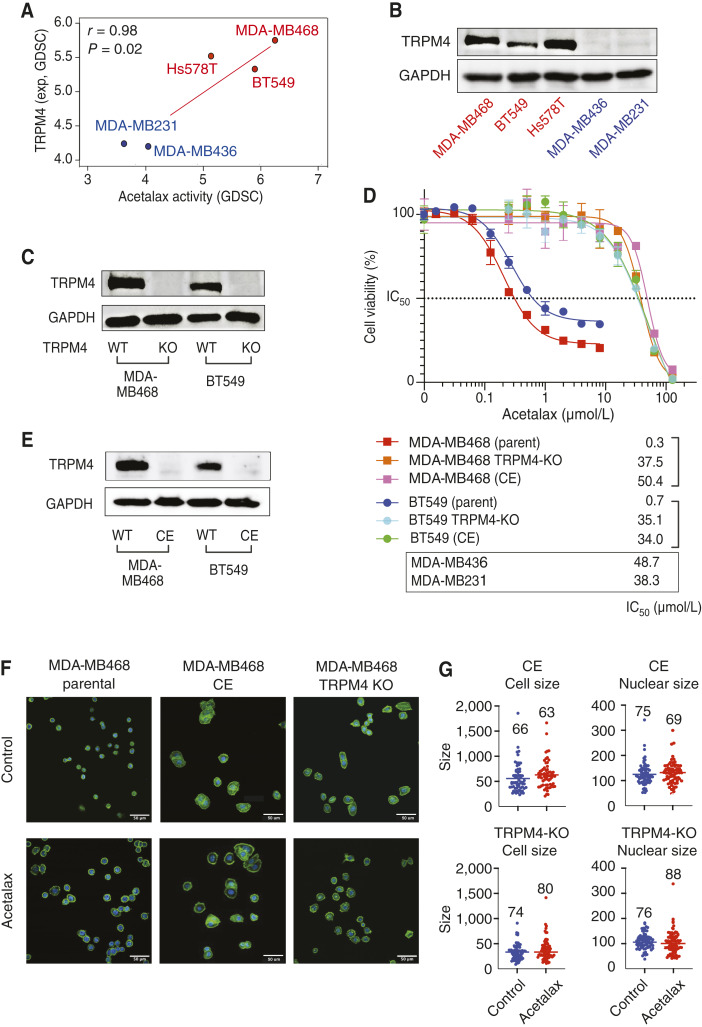
Role of TRPM4 in the action of Acetalax. **A,** Scatter plot demonstrating the correlation between Acetalax activity versus TRPM4 expression in the TNBC cell lines of the GDSC-MGH-Sanger database used in the present study (Supplementary Fig. S4 for the overall dataset with all cancer tissue types). The *x*-axis shows Acetalax activity. The *y*-axis shows the *TRPM4* transcript level measured by RNA-seq (log_2_). Both were visualized using CellMinerCDB. Each point is a TNBC cell line. The red line is the regression line. The Acetalax-sensitive cell lines are highlighted in red, and the resistant cell lines in blue. **B,** Western blots of TRPM4 levels in the five TNBC cell lines. GADPH was used as the loading control. **C,** Western blot of TRPM4 protein expression in MDA-MB468 and BT549 parental and TRPM4-KO cells. **D,** Acetalax cell viability assay in parental, TRPM4-KO, and CE cells. Cells were treated with Acetalax for 72 hours. Cell viability was determined using CellTiter-Glo 2. Experiments were performed in triplicate (*n* = 3). **E,** Western blot of TRPM4 expression in MDA-MB468 and BT549 parental and Acetalax-resistant (CE) cells. GADPH was used as the loading control. **F,** Representative microscopy images of parental MDA-MB468 cells and their resistant (CE) and TRPM4-KO counterparts. Cells were stained in blue with DAPI (binds DNA) and green with phalloidin (stains actin). Control, without Acetalax treatment. Scale bars, 50 μm. Cells were treated with Acetalax at 10 μmol/L for 30 minutes. Control, no drug. TRPM4 was degraded in the Acetalax-sensitive MDA-MB468 cells following Acetalax treatment for 4 hours. **G,** Quantification of repeated experiments as in **F**. Each cell and each nucleus are indicated by a dot. Numbers indicate the number of cells and nuclei analyzed.

Figure 4A demonstrates that the statistically significant correlation between Acetalax activity and TRPM4 transcript levels is also observed for the five TNBC cell lines used in the current molecular study. Moreover, Fig. 4B shows that the three Acetalax-sensitive TNBC cell lines, MDA-MB468, BT549, and Hs578T, have robust TRPM4 protein expression, whereas the resistant MDA-MB436 and MDA-MB231 cell lines show little to no expression.

To determine whether TRPM4 expression is causally linked with Acetalax response, we generated TRPM4-KO cell lines from MDA-MB498 and BT549 cells (Fig. 4C). When tested for cell killing by Acetalax, both KO cell lines showed marked drug resistance (Fig. 4D), consistent with the fact that TRPM4 expression determines the cellular cytotoxic response to Acetalax.

To further elucidate the relationship between Acetalax activity and TRPM4 expression, we also generated Acetalax-resistant clones by CE to increasing concentrations of Acetalax for 5 months using the Acetalax-sensitive MDA-MB468 and BT549 cells. Survival analyses in cells grown in Acetalax-free medium for 2 weeks demonstrated that both resistant (CE) cell lines showed approximately 50- to 100-fold resistance to Acetalax. Moreover, both cell lines showed comparable resistance level to the TRPM4-KO and TRPM4-nonexpressing cell lines (Fig. 4D) with quantification in lower part of the figure. Western blotting showed that both resistant cell lines had almost undetectable TRPM4 protein expression (Fig. 4E). However, TRPM4 transcripts remained unchanged in the resistant CE cell lines (Supplementary Fig. S5A), with the up- and downregulated pathways in response to Acetalax indicated in Supplementary Fig. S5B and S5C, respectively. To further implicate TRPM4 in the phenotypic response to Acetalax, we performed microscopy analyses, which showed that the TRPM4-KO and Acetalax-resistant cell lines failed to increase their overall size and nuclear size in response to Acetalax (Fig. 4F and G).

TRPM4 knock-in experiments were also done for the Acetalax-resistant, non–TRPM4-expressing MDA-MB231 and MDA-MB436 cell lines. Expression of TRPM4 protein was demonstrated, and gain of Acetalax sensitivity using cell viability assay was demonstrated (Supplementary Fig. S6).

Together, these results, including the correlation of TRPM4 gene and protein expression with Acetalax sensitivity and morphologic changes, the resistance of TRPM4-KO cells to Acetalax, and the downregulation of TRPM4 in cells with acquired resistance to Acetalax, implicate TRPM4 as a key molecular target of Acetalax.

### Acetalax induces TRPM4 degradation via the ubiquitin–proteasome system

**Figure 5:**
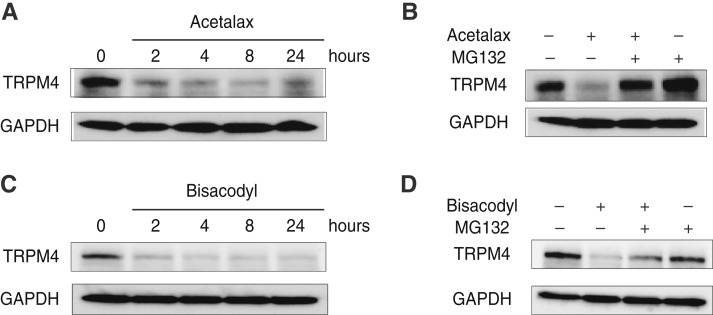
TRPM4 targeting and proteasomal degradation of TRPM4 by Acetalax and bisacodyl. **A,** Western blot of TRPM4 protein levels in MDA-MB468 following Acetalax treatment. **B,** Western blot of TRPM4 protein levels in MDA-MB468 following Acetalax and MG132 proteasome inhibitor treatment. **C,** Western blot of TRPM4 protein levels in MDA-MB468 following bisacodyl treatment. **D,** Western blot of TRPM4 protein levels in MDA-MB468 following bisacodyl and MG132 proteasome inhibitor treatment. The presence or absence of Acetalax or bisacodyl and MG132 is indicated in each experiment by + and/or − above each column. Cells were treated with Acetalax for 4 hours at 10 μmol/L and bisacodyl at 30 μmol/L. Cells were treated with MG132 for 4 hours at 20 μmol/L.

Because cellular macromolecule targets that are poisoned by drugs, such as topoisomerases and DNA methyltransferases, tend to be degraded to minimize the drug effects (23), we tested the effects of Acetalax on cellular TRPM4 protein. Figure 5A shows the effects of Acetalax on TRPM4 protein expression. It demonstrates that Acetalax decreases TRPM4 expression by 4 hours in a time-dependent manner.

The decrease in TRPM4 by Acetalax was blocked by the proteasome inhibitor MG132 (Fig. 5B), indicating that Acetalax induces TRPM4 degradation via the ubiquitin–proteasome system. Similar results were observed for bisacodyl (Fig. 5C and D), which leads us to conclude that both Acetalax and bisacodyl poison TRPM4 to kill cancer cells.

## Discussion

**Figure 6:**
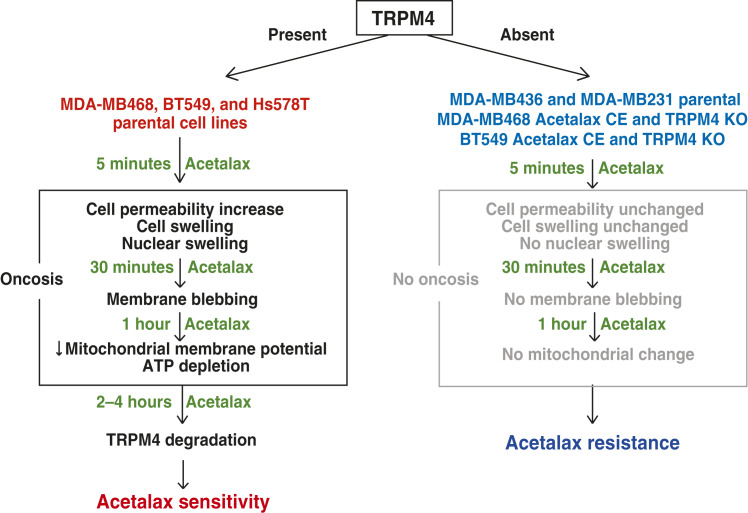
Flow chart. **A,** Schematic providing a synopsis of the effect of TRPM4 protein presence or absence on Acetalax response in TNBCs.

In the current study, we demonstrate that Acetalax and bisacodyl cause oncosis and induce the degradation of the cell membrane protein TRPM4 in Acetalax-sensitive TNBC cells (Fig. 6). Acetalax-resistant TNBC cells did not express TRPM4 or undergo oncosis. Moreover, Acetalax-resistant TNBC cells generated by CE to Acetalax also did not express TRPM4, leading to Acetalax resistance. TRPM4-KO TNBC cells were also generated. We found that these cells lacked TRPM4 expression and oncosis induction, as they gained Acetalax resistance. We also conducted TRPM4 knock-in experiments in the two Acetalax-resistant lines, demonstrating the gain of Acetalax sensitivity (Supplementary Fig. S6). Thus, we discovered that TRPM4 expression is required for Acetalax and bisacodyl sensitivities in TNBC cells by inducing oncosis, whereas the lack of TRPM4 expression causes Acetalax resistance. Oncosis events occur within minutes after Acetalax administration with increased cell permeability, cytosolic and nuclear swelling, appearance of cell membrane blebbing, followed by a decrease in mitochondrial membrane potential, and ATP depletion. In addition, loss of TRPM4 expression occurred, and mitochondrial fragmentation was induced by 4 hours. Figure 6 illustrates a synopsis of the stark differences in the responses of the TNBC cell lines to Acetalax dependent on the presence or absence of the TRPM4 protein.

Various mechanistic classifications of cell death have been reported. Some classify cell death morphologically, whereas others focus on biochemical alterations. Oncosis was originally defined as a form of cell death accompanied by cell swelling and later reported to be associated with energy depletion, increased cell membrane permeability, cell membrane blebbing, and mitochondrial dysfunctions (12, 24). Some reports classify oncosis as one pathway of programmed necrosis. However, this has not been widely accepted yet (20, 24). From our investigations, we propose that Acetalax-induced cell death is caused by oncosis. Indeed, Acetalax induced cell swelling in Acetalax-sensitive TNBC cells (Fig. 2), and increased cell membrane permeability occurred concurrently with cell swelling (Fig. 3), which is essential for oncosis-associated cell death (12), implicating linkage between the two. Acetalax was shown to result in additional features of oncosis, including membrane blebbing, mitochondrial dysfunction, and ATP depletion (Figs. 2C and D, 3B–D; refs. 12, 13, 25). Additionally, our data show a time lag between the initial and rapid (5 minutes) events prior to the decrease in mitochondrial membrane potential and ATP depletion, suggesting that these early events of increased membrane permeability lead to mitochondrial dysfunction, ATP depletion, and mitochondrial fragmentation.

We also studied mitochondrial morphologic changes, in particular mitochondrial fragmentation that occurs 4 hours or later after Acetalax exposure. Mitochondria undergo constant changing in their morphology through fusion and fission, known as mitochondrial dynamics, which is important for mitochondrial function and maintenance (26). Mitochondrial fusion contributes to the maintenance of total mitochondrial function by allowing multiple mitochondria to share the DNA and proteins that construct mitochondria (27). Mitochondrial fission, on the other hand, is known to selectively remove damaged mitochondria, leading to autophagy (28). Mitochondrial fragmentation occurs when mitochondrial dynamics are out of balance and fission is enhanced, resulting in cytochrome C release and apoptosis (29). We observed mitochondrial fragmentation at 4 hours after Acetalax administration (Fig. 3D), indicating that mitochondrial fragmentation occurred after oncosis (Fig. 6).

Cell swelling did not occur in TRPM4-KO cells or in cells chronically exposed to Acetalax, leading to suppression of TRPM4 expression (Fig. 4F and G). TRPM4-KO cells were Acetalax-resistant (Figs. 1B and 4G), and conversely, TRPM4-transfected cells were sensitized to Acetalax (Supplementary Fig. S6A and S6B), implicating the role of TRPM4 in Acetalax-induced oncosis. These results imply that TRPM4 can be a treatment response predictor of Acetalax, which is clinically relevant for the drug development of Acetalax as a prospective anticancer agent. Thus, TRPM4 may serve as a potential predictor for patients with TNBC who may respond to Acetalax, and future clinical trials should test this hypothesis. Notably, in two recent studies, TRPM4 expression was reported to be upregulated in breast cancer samples at both the mRNA and protein levels (22, 30, 31).

The clear difference of responses to Acetalax for multiple phenotypic, morphologic, and biochemical factors dependent on the presence or absence of the TRPM4 protein shown in Fig. 6 indicates oncosis occurrence when TRPM4 is present in these TNBC cell lines and also that TRPM4 is a dominant biomarker for Acetalax activity.

To further elucidate the downstream effects of Acetalax, we performed RNA-seq in MDA-MB468 and BT549 cells treated with Acetalax for 6 hours. Supplementary Fig. S5B shows several gene sets statistically significantly enriched in both the Acetalax-sensitive MDA-MB468 and BT549 cells. They include the clinically relevant inflammatory and associated immune responses, as well as immediate early response, EMT, hypoxia, and unfolded protein response. Conversely, downregulated gene sets include multiple cell cycle– and cell proliferation–related gene categories. These results are consistent with the induction of an innate immune response and block of cell proliferation by Acetalax-induced oncosis. The EMT gene set enrichment is consistent with prior results showing that the epithelial–mesenchymal signature had the strongest correlation with Acetalax activity of 297 drugs tested and indicated generally better activity in the epithelial cell lines (5, 16).

We are aware that our study has limitations. It focuses on the elucidation of the molecular pharmacology of the diphenol laxatives Acetalax and bisacodyl as potential anticancer agents. We did not perform any xenograft studies beyond those published (2), and oncosis remains a relatively poorly defined process at the molecular level. Our findings raise new prospects for elucidating the molecular pharmacology of diphenol laxatives. Until now, aquaporins (AQP3) and sodium–potassium ATPases have been involved as molecular targets of bisacodyl in studies performed many years ago, but the molecular target or targets of diphenol laxatives have remained elusive (32–35). Whether TRPM4 targeting contributes to the laxative activity of diphenol laxatives warrants further studies, which are beyond the realm of the current report that concentrates on the potential anticancer activity of Acetalax and bisacodyl in TNBC.

## Supplementary Material

Supplementary Figure 1 Bisacodyl structure, effect on TNBC cell lines and similarity to Acetalax.

Supplementary Figure 2 Morphological changes in TRPM4-KO and chronic exposure (CE) cells.

Supplementary Figure 3 Mitochondrial morphological changes caused by Acetalax in BT549.

Supplementary Figure 4 Scatter plot of Acetalax activity versus TRPM4 transcript expression.

Supplementary Figure 5 TRPM4 RNA expression and categories of gene sets enriched in response to Acetalax treatment.

Supplementary Figure 6 Exogenous TRPM4 expression sensitizes TRPM4 negative and acetalax-resistant MDAMB231 and MDAMB436 cells to acetalax.

Supplementary Figure Legends 1-6 Legends for the Supplementary figures.
