# Supplementary figures and images for: Acetalax (Oxyphenisatin Acetate, NSC 59687) and Bisacodyl Cause Oncosis in Triple-Negative Breast Cancer Cell Lines by Poisoning the Ion Exchange Membrane Protein TRPM4
Source: Cancer Res Commun. 2024 Aug 14;4(8):2101–11. doi: 10.1158/2767-9764.CRC-24-0093 (PMC11322923; doi:10.1158/2767-9764.CRC-24-0093)

Supplemental Figure 1

A

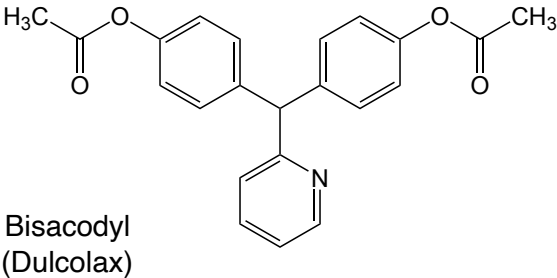

B

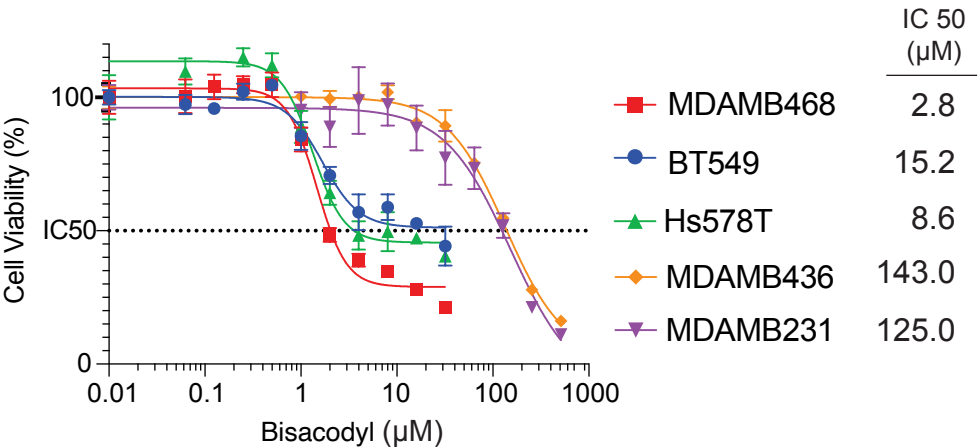

C

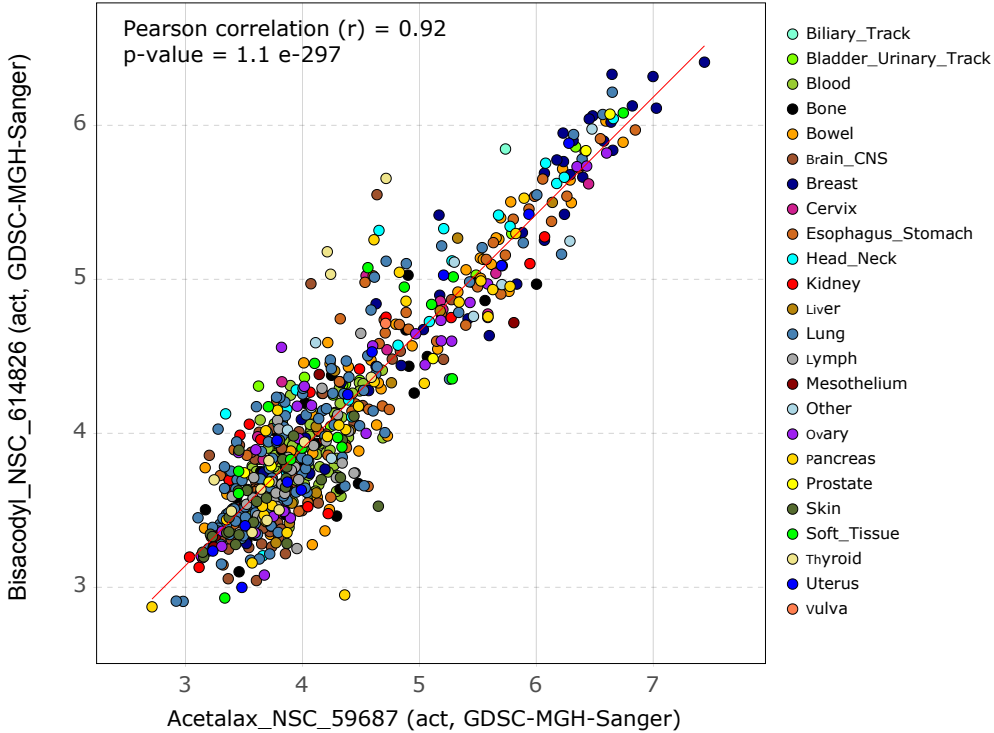

D

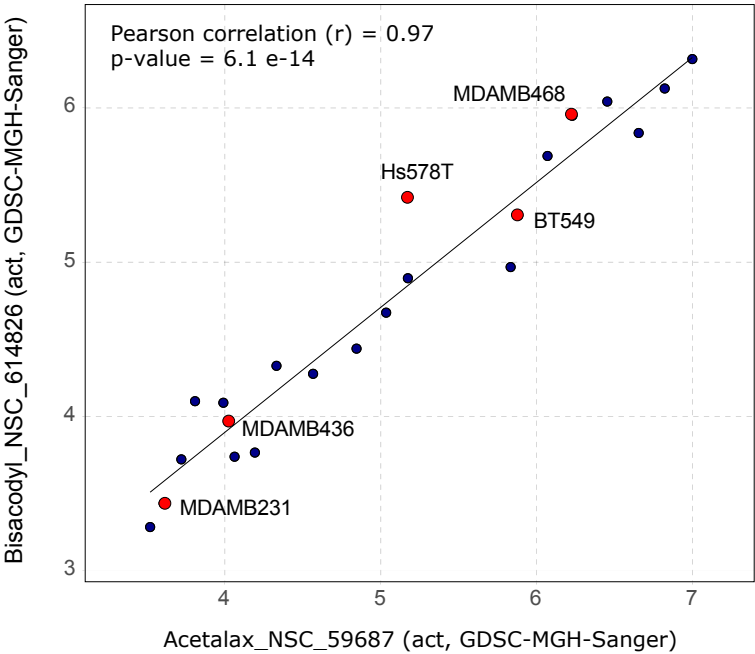

Supplement: Supplementary Figure 1 — Bisacodyl structure, effect on TNBC cell lines and similarity to Acetalax. [file crc-24-0093_supplementary_figure_1_suppsf1.pdf]

Supplemental Figure 2

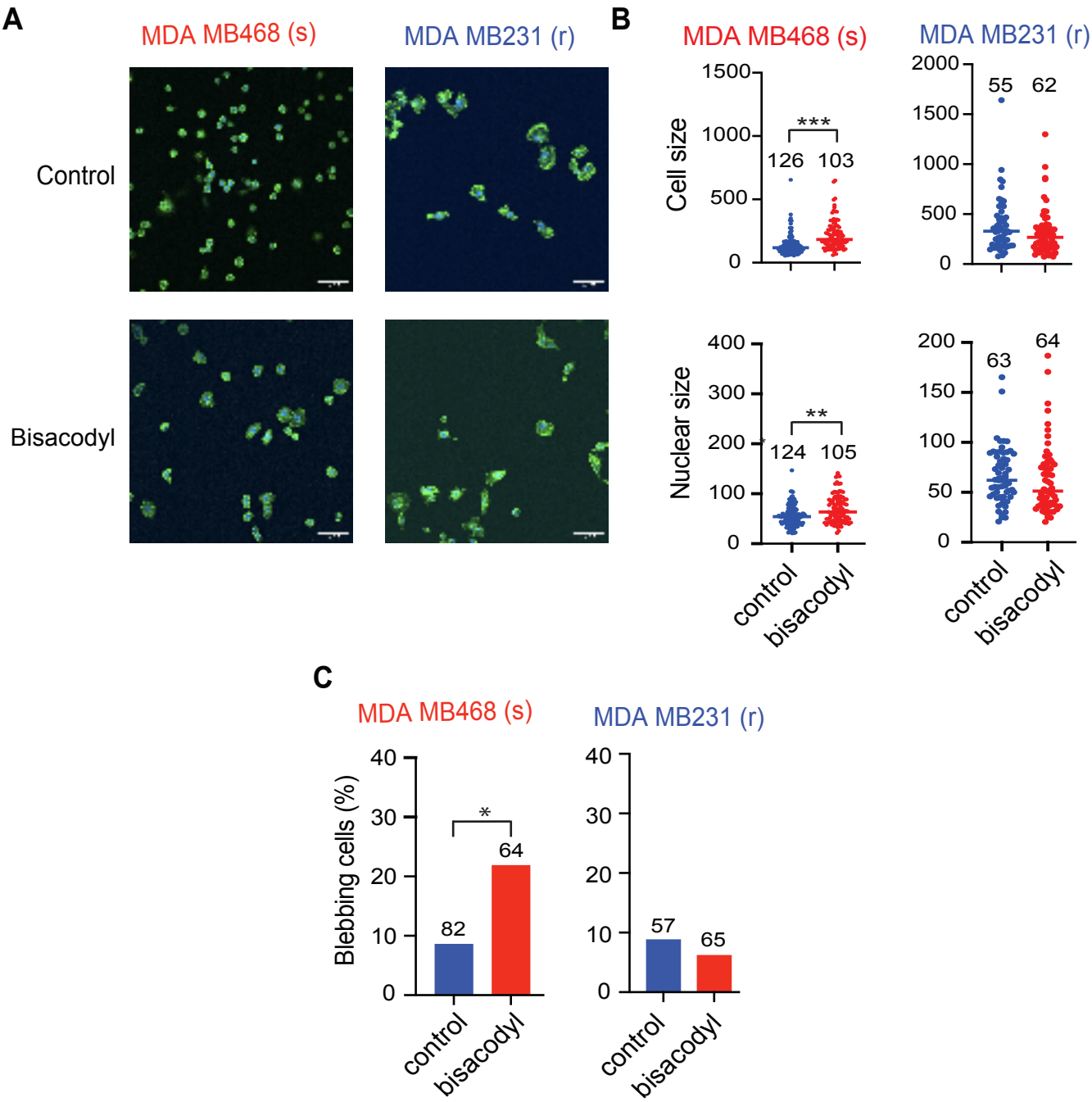

Supplement: Supplementary Figure 2 — Morphological changes in TRPM4-KO and chronic exposure (CE) cells. [file crc-24-0093_supplementary_figure_2_suppsf2.pdf]

## Supplemental Figure 3

BT549 (S) control

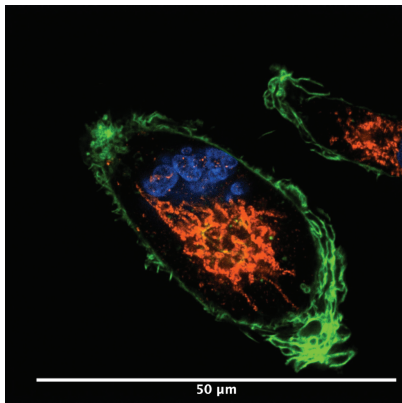

BT549 (S) Acetalax 10uM 4h

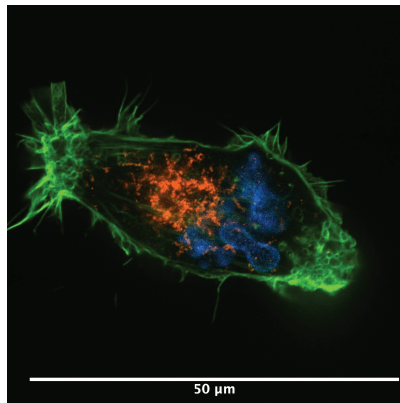

Supplement: Supplementary Figure 3 — Mitochondrial morphological changes caused by Acetalax in BT549. [file crc-24-0093_supplementary_figure_3_suppsf3.pdf]

Supplemental Figure 4

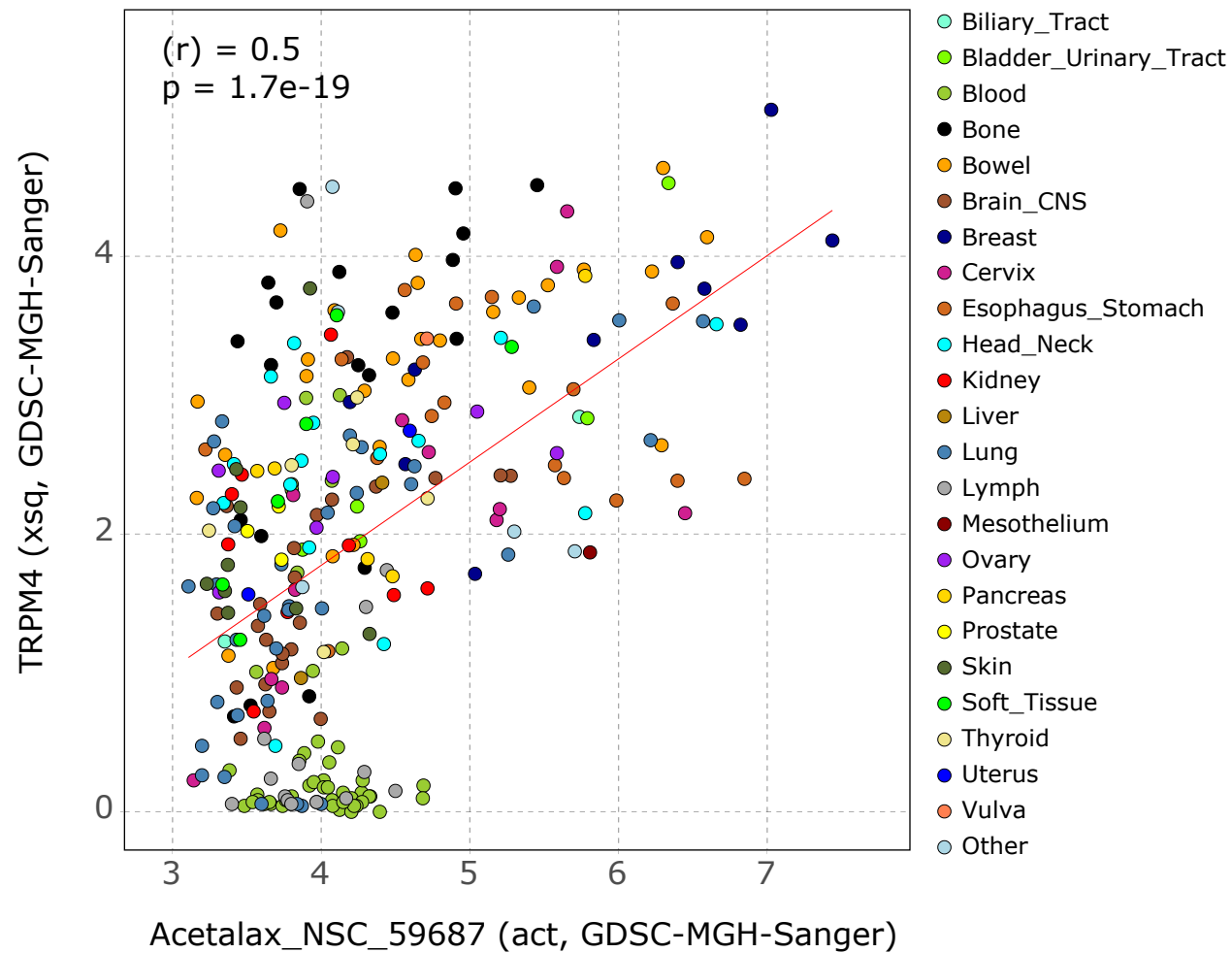

Supplement: Supplementary Figure 4 — Scatter plot of Acetalax activity versus TRPM4 transcript expression. [file crc-24-0093_supplementary_figure_4_suppsf4.pdf]

## Supplemental Figure 6

**A**

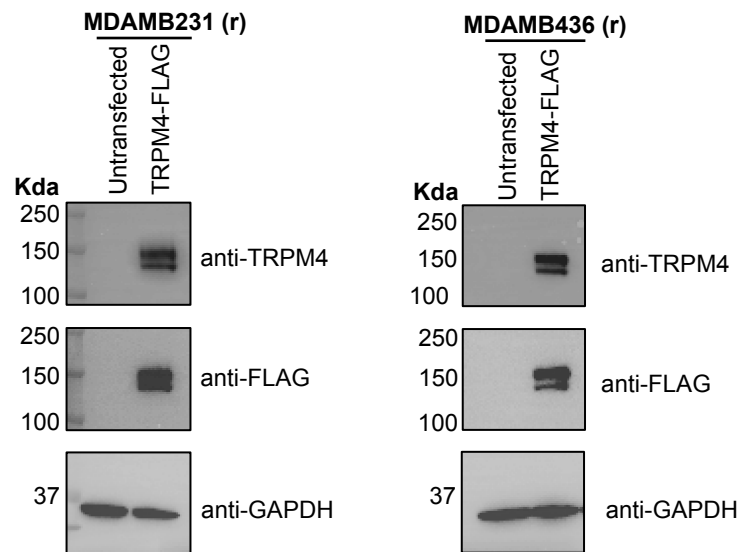

**B**

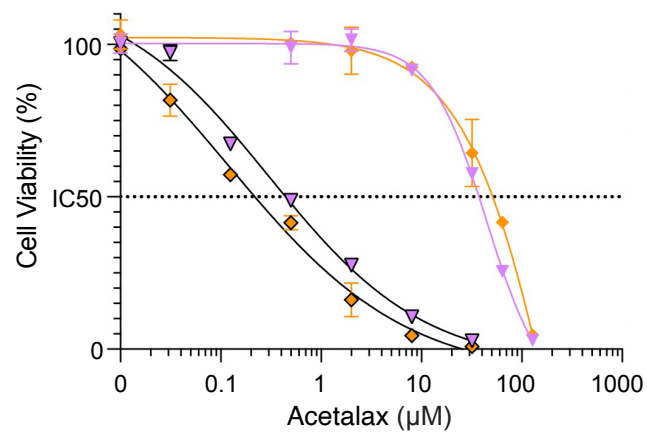

|                      | IC50 uM |
|----------------------|---------|
| MDAMB231 (r)         | 31.5    |
| MDAMB231 + TRPM4 (s) | 0.47    |
| MDAMB436 (r)         | 41.8    |
| MDAMB231 + TRPM4 (s) | 0.24    |

Supplement: Supplementary Figure 6 — Exogenous TRPM4 expression sensitizes TRPM4 negative and acetalax-resistant MDAMB231 and MDAMB436 cells to acetalax. [file crc-24-0093_supplementary_figure_6_suppsf6.pdf]
