# Supplementary material for: Acetalax (Oxyphenisatin Acetate, NSC 59687) and Bisacodyl Cause Oncosis in Triple-Negative Breast Cancer Cell Lines by Poisoning the Ion Exchange Membrane Protein TRPM4
Source: Cancer Res Commun. 2024 Aug 14;4(8):2101–11. doi: 10.1158/2767-9764.CRC-24-0093 (PMC11322923; doi:10.1158/2767-9764.CRC-24-0093)
Supplement: Supplementary Figure 5 — TRPM4 RNA expression and categories of gene sets enriched in response to Acetalax treatment. [file crc-24-0093_supplementary_figure_5_suppsf5.pdf]

Supplemental Figure 5

A

RNA sequence expression of TRPM4

Gene \ sample	MDAMB468 Parent control	MDAMB468 Parent Acetalax	MDAMB468 CE control	BT549 Parent control	BT549 Parent Acetalax	BT549 CE control
TRPM4	2.566	2.691	2.825	1.771	1.406	2.382

B

Upregulated pathways in response to Acetalax

MDAMB468 control vs acetalax	BT549 control vs acetalax	Pathway enhanced by acetalax by normalized enrichment scores
2.31	2.25	IER Signature
2.36	2.19	TNFA SIGNALING VIA NFKB
1.72	1.29	TGF BETA SIGNALING
1.85	1.20	IL2 STAT5 SIGNALING
2.04	1.67	INFLAMMATORY RESPONSE
1.56	0.91	IMMUNE SIGNATURE
1.69	1.05	IL6 JAK STAT3 SIGNALING
2.17	1.51	EPITHELIAL MESENCHYMAL TRAN
1.96	1.75	HYPOXIA
1.37	1.38	UNFOLDED PROTEIN RESPONSE

C

Downregulated pathways in response to Acetalax

MDAMB468 control vs Acetalax	BT549 control vs acetalax	Pathway reduced by acetalax by normalized enrichment scores
-2.16	-1.78	MYC TARGETS V1
-1.96	-1.98	E2F TARGETS
-1.93	-1.88	G2M CHECKPOINT
-1.89	-1.85	MYC TARGETS V2
-1.33	-1.05	DNA REPAIR
-0.99	-1.49	MITOTIC SPINDLE
-0.84	-1.12	WNT BETA CATENIN SIGNALING
