# Supplementary material for: Acetalax (Oxyphenisatin Acetate, NSC 59687) and Bisacodyl Cause Oncosis in Triple-Negative Breast Cancer Cell Lines by Poisoning the Ion Exchange Membrane Protein TRPM4
Source: Cancer Res Commun. 2024 Aug 14;4(8):2101–11. doi: 10.1158/2767-9764.CRC-24-0093 (PMC11322923; doi:10.1158/2767-9764.CRC-24-0093)
Supplement: Supplementary Figure Legends 1-6 — Legends for the Supplementary figures. [file crc-24-0093_supplementary_figure_legends_1-6_suppsf1-sf6.docx]

**Acetalax (Oxyphenisatin acetate, NSC 59687) and Bisacodyl Cause Oncosis in Triple Negative Breast Cancer by Poisoning the Ion Exchange Membrane Protein TRPM4**

Makito Mizunuma^1^, Christophe E. Redon^1^, Liton Kumar Saha^1^, Andy D. Tran^2^, Anjali Dhall^1^, Robin Sebastian^1^, Michael J. Kruhalk^2^, William C, Reinhold^1§^, Naoko Takebe^1.3§^, Yves Pommier^1§*^

^1^ Developmental Therapeutics Branch, Center for Cancer Research, National Cancer Institute, National Institutes of Health, Bethesda, MD, USA

^2^ Laboratory of Cancer Biology and Genetics, Center for Cancer Research, National Cancer Institute, National Institutes of Health, Bethesda, MD, USA

^3^ Division of Cancer Treatment and Diagnosis, National Cancer Institute, National Institutes of Health, Bethesda, MD, USA

§ Co-senior authors

* Corresponding author

Running Title: Acetalax Causes Oncosis via TRPM4

Keywords: Acetalax, Drug Repurposing, TNBC, Oncosis, TRPM4, Cell Swelling, Biomarker, Therapeutics

**Corresponding Authors:** Yves Pommier, Developmental Therapeutics Branch, CCR/NCI, NIH. Building 37, Room 5068 Bethesda, MD 20892-4255, USA

Phone: 240-760-6142

E-mail: [pommier@nih.gov](mailto:pommier@nih.gov)

**Supplemental Figures**


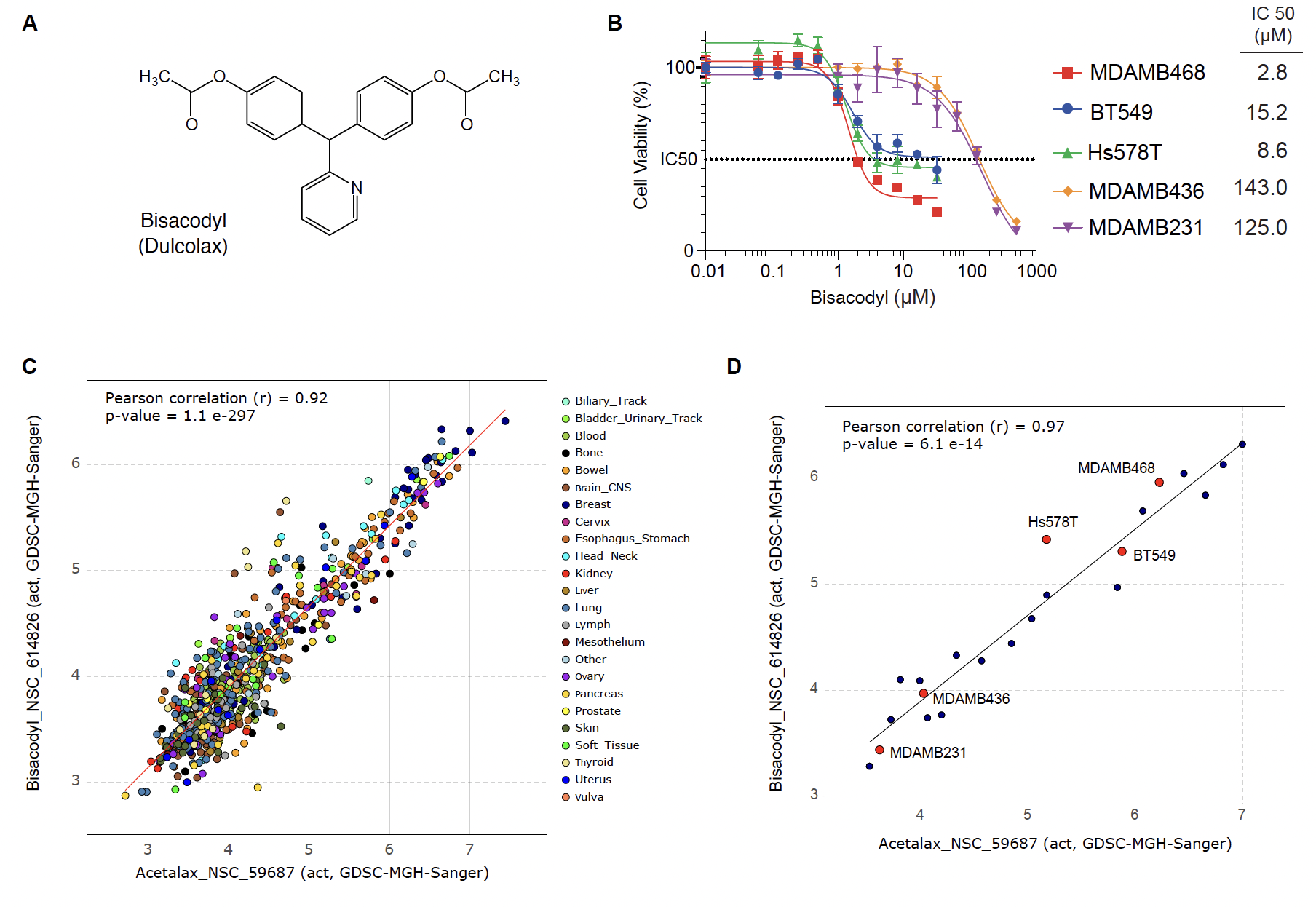


**Supplemental Figure 1: Bisacodyl structure, effect on TNBC cell lines and similarity to Acetalax.**

**A.** Two-dimensional structure of Bisacodyl (NSC614826). **B.** Cell viability assays for five TNBC cells measured at 72 hours using CellTiter-Glo 2. Experiments were performed in triplicate (n=3). Bars represented Standard Deviations. **C.** Scatter plot of GDSC-MGM-Sanger drug activity (-log10[IC50M]) of Acetalax (x-axis) versus Bisacodyl (y-axis) for all tisues of origin (713 cell lines). The red line is the regression line. Each circle is a cell line. **D.** Scatter plot of GDSC-MGM-Sanger drug activity (-log10[IC50M]) of Acetalax (x-axis) versus Bisacodyl (y-axis) for 22 triple negative breast cancer cell lines. The red line is the regression line. Each circle is a cell line. The red circles are the cell lines focused on for the current study. Visualization for **C** and **D** are done using CellMinerCDB (<https://discover.nci.nih.gov/rsconnect/cellminercdb/>).


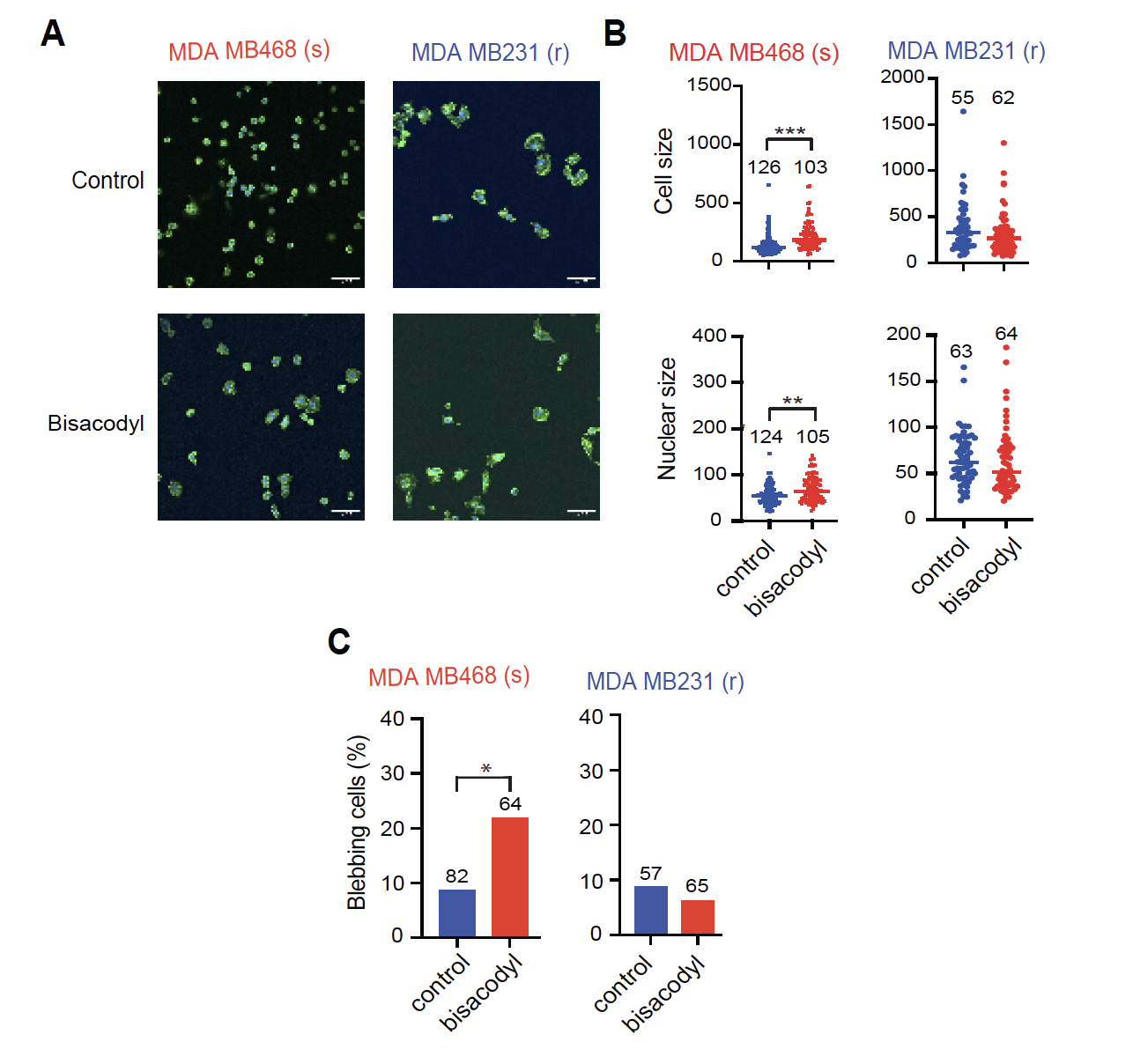


**Supplemental Figure 2: Morphological changes in TRPM4-KO and chronic exposure (CE) cells.**

**A.** Representative microscopy images of cell lines treated with Bisacodyl (30 μM for 30 min) and stained in blue with DAPI (binds DNA) and green with phalloidin (stains actin). Control row is without Bisacodyl treatment. Scale bars indicate 50 μm. (s) indicates the most sensitive and (r) an Acetalax resistant cell lines (see Figure 1B). **B.** Violin plot quantitation of Bisacodyl-induced cell and nuclear size increase at 30 minutes. The lines indicate medians. Each dot is a cell. MDAMB468 has significant changes in cell size. **C.** Quantitation of membrane blebbing in multiple cells. In the most sensitive (s) cell line MDAMB468, Bisacodyl treatment significantly increased the frequency of blebbing. Statistical significance is indicated by * for p<0.005, ** for p<0.0005 and *** for p<0.0001 (by Mann-Whitney U-test, calculated in PRISM 9.0).


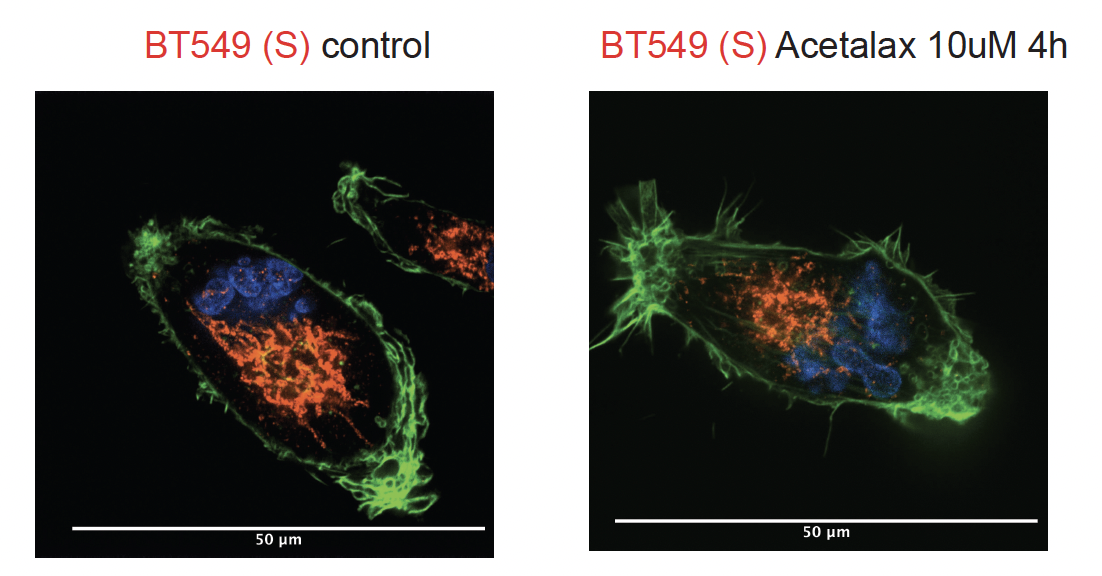


**Supplemental Figure 3: Mitochondrial morphological changes caused by Acetalax in BT549.**

Representative microscopy images of BT549 treated with Acetalax (10 μM for 4 hours) and stained in blue with DAPI (binds DNA), green with phalloidin (stains actin) and red with TOMM20 (stains mitochondria). Control is without Acetalax treatment. Scale bars indicate 50 μm.

**
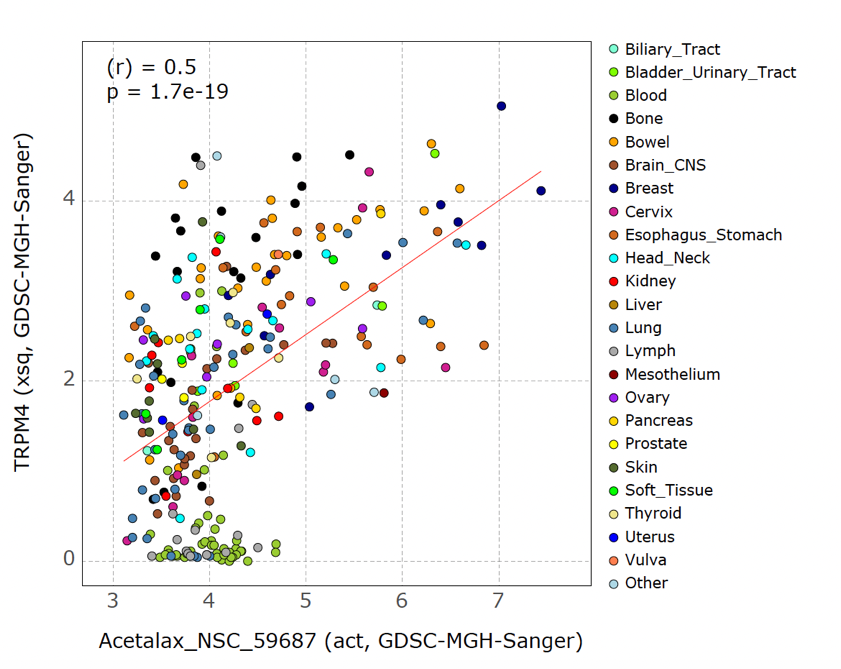
**

**Supplemental Figure 4: Scatter plot of Acetalax activity versus TRPM4 transcript expression.**

Scatter plot demonstrating the correlation between Acetalax activity (x-axis) and TRPM4 transcript expression (y-axis) in all 286 cell lines (with data) of the GDSC-MGH-Sanger database. The x-axis is Acetalax activity expressed as (-log10[IC50M]). The y-axis is *TRPM4* transcript level measured by RNAseq (log2). Visualization is done using CellMinerCDB (<https://discover.nci.nih.gov/rsconnect/cellminercdb/>). Each circle is a cell line. The red line is the regression line.


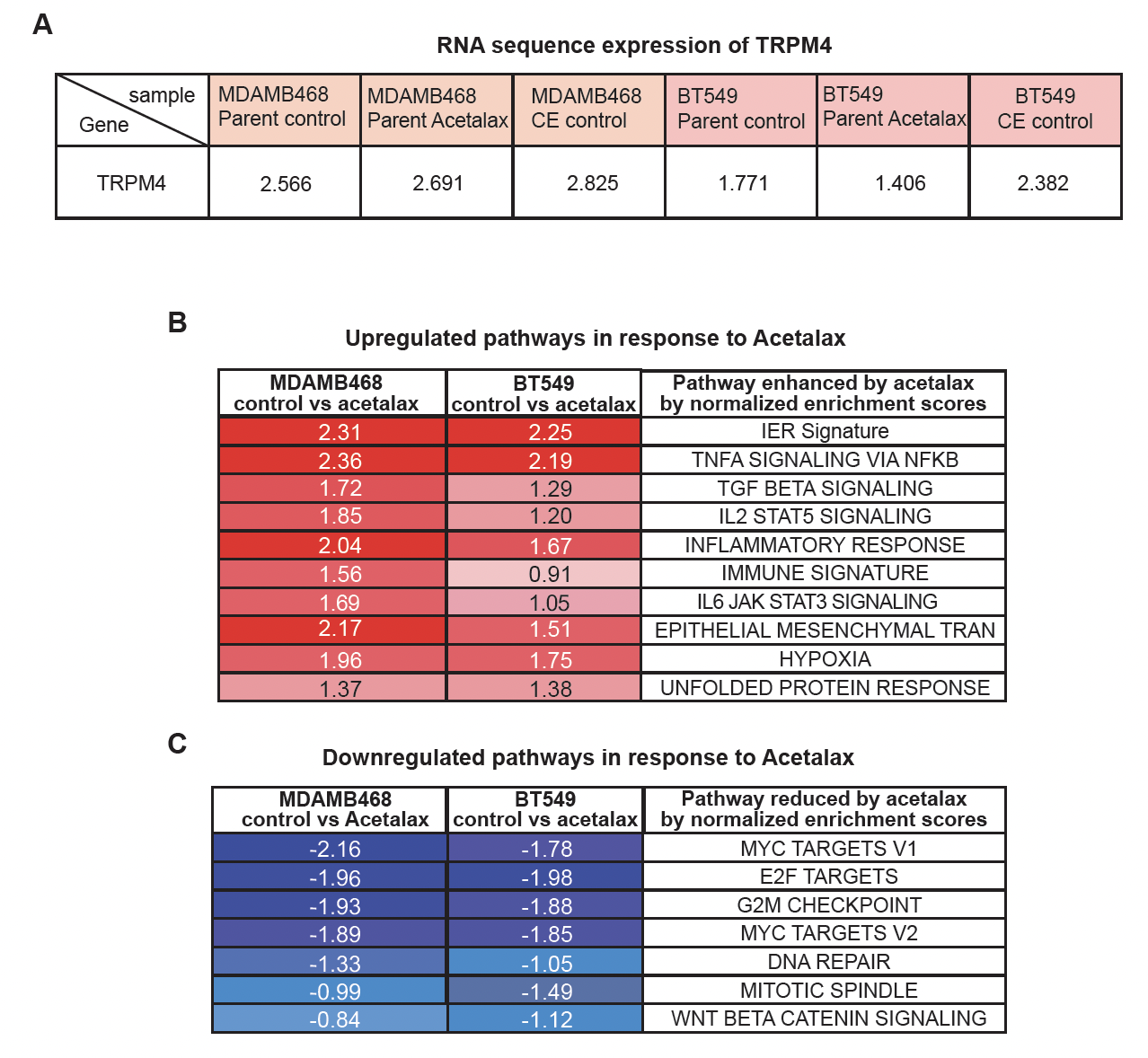


**Supplemental Figure 5: TRPM4 RNA expression and categories of gene sets enriched in response to Acetalax treatment.**

**A.** RNA-seq log2(FPKM+1) values for TRPM4 gene in control (no drug) and acetalax at 1 μM for 6 hours treatment in MDAMB468 and BT549 parental cells, and chronically exposed cells (CE) control (no drug) in MDAMB468 and BT549 cells. **B.** Gene sets upregulated by acetalax treatment in both cell lines. MDAMB468 and BT549 parent cells in no-drug (control) compared to acetalax 1 μM for 6 hours treatment. Each gene’s score is computed by taking the difference between treatment and control for each condition. Positive and negative scores represent up and down regulation of genes, respectively. Enrichment analysis was performed using GSEApy python library. We used 50 Hallmark gene sets, EMT gene sets, Immediate early response (IER) genes and immune signatures in the enrichment analysis. **C.** Gene sets downregulated by acetalax treatment in both cell lines. Analysis was carried out as described in panel B.


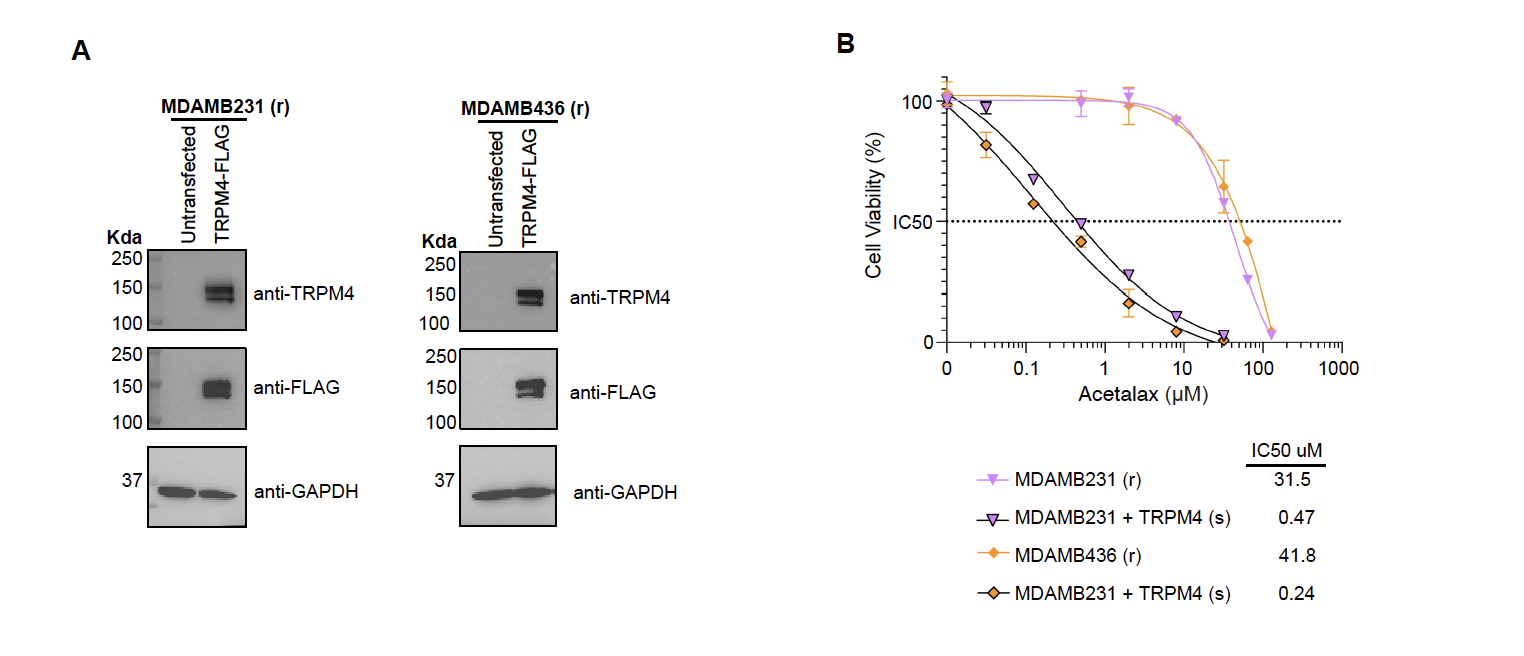


**Supplemental Figure 6:**

Exogenous TRPM4 expression sensitizes TRPM4 negative and acetalax-resistant MDAMB231 and MDAMB436 cells to acetalax. **A.** Western blots of MDAMB231 (left) and MDAMB436 (right) cells transfected with FLAG-tagged TRPM4 plasmid for 72 h probed with anti-TRPM4 and anti-FLAG antibodies. GAPDH was used as loading control. **B.** Cell viability assay of untransfected and FLAG-tagged TRPM4-transfected MDAMB231 and MDAMB436 cells. The x-axis is the concentration of acetalax added and the y-axis the percentage of surviving cells.The cell viability was subsequently done at 72 hours post acetalax treatment. The measured IC50 is included. Error bars represent the mean ± SD of three independent experiments.
